# Decision Tree Approach to the Impact of Parents’ Oral Health on Dental Caries Experience in Children: A Cross-Sectional Study

**DOI:** 10.3390/ijerph15040692

**Published:** 2018-04-06

**Authors:** Shinechimeg Dima, Kung-Jeng Wang, Kun-Huang Chen, Yung-Kai Huang, Wei-Jen Chang, Sheng-Yang Lee, Nai-Chia Teng

**Affiliations:** 1 School of Dentistry, College of Oral Medicine, Taipei Medical University, 250 Wu-Hsing Street, Taipei 110, Taiwan; d204103003@tmu.edu.tw (S.D.); cweijen1@tmu.edu.tw (W.-J.C.); seanlee@tmu.edu.tw (S.-Y.L.); 2 Department of Industrial Management, National Taiwan University of Science and Technology, No. 43, Section 4, Jilong Road, Da’an District, Taipei 10607, Taiwan; kjwang@mail.ntust.edu.tw; 3 Big Data Research Center, Asia University, Lioufeng Rd. 500, Wufeng, Taichung 41354, Taiwan; khchen@mail.ntust.edu.tw; 4 School of Oral Hygiene, College of Oral Medicine, Taipei Medical University, 250 Wu-Hsing Street, Taipei 110, Taiwan; ykhuang@tmu.edu.tw; 5 Department of Dentistry, Taipei Medical University Hospital, 250 Wu-Hsing Street, Taipei 110, Taiwan

**Keywords:** early childhood caries, periodontitis, decision tree classification

## Abstract

Decision tree (DT) analysis was applied in this cross-sectional study to investigate caries experience in children by using clinical and microbiological data obtained from parent–child pairs. Thirty pairs of parents and children were recruited from periodontal and pediatric dental clinics. All participants were clinically examined for caries and periodontitis by a calibrated examiner. Cariogenic and periodontopathic bacteria examinations were conducted. The Kendall rank correlation coefficient was used to measure the association between data variables obtained through clinical and microbiological examinations. A classificatory inductive decision tree was generated using the C4.5 algorithm with the top-down approach. The C4.5 DT analysis was applied to classify major influential factors for children dental caries experience. The DT identified parents’ periodontal health classification, decayed, missing, filled permanent teeth (DMFT) index, periodontopathic test (PerioCheck) result, and periodontal pocket depth as the classification factors for children caries experience. 13.3% of children were identified with a low decayed, missing, filled primary teeth (dmft) index (dmft < 3) whose parents had a periodontal pocket depth ≤3.7, PerioCheck score >1, DMFT index <13.5, and periodontal classification >2. The DT model for this study sample had an accuracy of 93.33%. Here, parental periodontal status and parents’ DMFT were the factors forming the DT for children’s caries experience.

## 1. Introduction

The prevalence of dental caries in preschool children in Taiwan was 79.3% in 2011. Caries risk assessment in children is a crucial part of the clinical decision-making process that dentists apply on a daily basis to provide appropriate preventive measures. Although previous caries experience may be the most informative criterion and a basis for assessing caries risk [1], from a clinical standpoint, the information comes out too late to be useful in preventing caries, as many irrevocable changes have already occurred [2]. Previous studies have investigated parental factors associated with caries in children [3,4,5,6], focusing on the social determinants of the parents [4] and parenting behaviors and practices [3].

Caries development in preschool children has been associated with the family’s socioeconomic situation and oral health behavior [7]. The association between low health literacy and poor health outcomes is well established [8]. Patients with low oral health literacy (OHL) are at the highest risk for oral diseases and problems [9]. Further, low health literacy may be associated with barriers to accessing care and with oral health behaviors such as seeking preventive care [10].

Lack of time for implementing caries risk assessments in clinical practice is another challenge. Regular oral examinations and biometrics tests with simple kits are easily used tools in dentists’ daily practice. To maintain optimal oral health, the American Dental Association (ADA) recommends regular dental visits, at intervals determined by a dentist [11]. Regular dental exams are an important part of preventive oral health care. During a dental visit, professional tooth cleaning and screening for cavities and gum disease are routine procedures. Parental oral health and beliefs usually represent their own clinical outcome [12], and it is associated with the oral health status of their children [13].

Data mining is an effective and practical statistical method for identifying crucial associations in data obtained from various perspectives. The decision tree (DT) model is a powerful and nonparametric statistical method commonly used in data mining to examine complex data and induce the DT, which is used to make classifications or predictions [14]. In dental research, the DT model has been applied to predict primary and secondary caries risk factors in an adult population [15]. Unlike traditional regression model analysis–which uses multiple factors and longitudinal data–the DT model does not require assumptions about the predictor variables for the outcome or the presence or absence of interactions among the predictor variables. The decision trees generated by the C4.5 algorithm can be used for classification, and for this reason, C4.5 is often referred to as a statistical classifier.

The objective of this study was to apply DT analysis to investigate caries experience in children by using clinical and microbiological data obtained from parent–child pairs. The hypothesis we tested was that the DT approach can find a pilot model of caries experience in preschool children through their own and parents’ dental caries and periodontal health status examined clinically and microbiologically.

## 2. Materials and Methods

### 2.1. Study Participants and Study Design

A simple random sampling process was conducted in one center in Taipei. In this study, 30 pairs of parents and children were recruited from periodontal and pediatric dental clinics. Children participants were between 3 and 7 years of age (3–4 years, *N* = 12; 5 years, *N* = 10; 6–7 years, *N* = 8). Nineteen were girls (63.3%) and 11 were boys (36.7%). The mean scores of the decayed, missing, filled permanent teeth (DMFT) and primary teeth (dmft) indices were 12.90 and 8.87 in the parents and children, respectively [5]. Before conducting the study, ethical approval and informed consent from participants were obtained. The inclusion criteria for parents were being the main caregiver of a child, absence of any medical history associated with systemic or chronic diseases, and no major dental history except dental caries. The children were required to present complete primary or mixed dentition with only the first permanent molars erupted. The study and methods were carried out in accordance with the guidelines of the Declaration of Helsinki (DoH) and approved by full-board review process of the Taipei Medical University-Joint Institutional Review Board. The dataset does not contain any direct identifiers. Datasets on which the conclusions of the manuscript rely are presented in the main paper. If additional supporting files are needed, the dataset is available per editor’s request.

### 2.2. Clinical Examination

All 30 parent–child pairs were recruited for dental examination by a trained dentist in a fully equipped dental unit in Taipei Medical University Hospital during 2005. World Health Organization (WHO) diagnostic criteria for dental caries and periodontal health were used to evaluate the dental caries in parent–child pairs and periodontal status in parents, respectively. Following thorough clinical examination by using a probe, cotton roll, and mirror, DMFT and dmft were calculated in parents and children, respectively. In this study we used dmft, since the exfoliation primary teeth were not counted. Community Periodontal Index (CPI) is a simple screening method used worldwide [16,17], which evaluates treatment needs of populations and individuals. The examination of the subjects in this study was conducted according to WHO guidelines using the WHO CPITN–E probe. The teeth examined were 17, 16, 11, 26, 27, 36, 37, 31, 46 and 47. Scoring criteria were followed as previously suggested. According to their periodontal health status, parents were classified into grades from 0 to 4, on the basis of the severity of periodontal disease and treatment needs. To evaluate oral hygiene in participants, the Plaque Control Record (PCR) developed by O’Leary [18] was applied after all of the teeth were examined and scored using Trace^@^ Plaque Disclosing Liquid (Young Dental Manufacturing, Earth City, MO, USA). 

### 2.3. Microbiological Examination

Oral bacteria tests examining the growth of *Streptococcus mutans* and periodontal pathogens were collected from participants by using the Dentocult SM dip strip (Orion Diagnostica, Espoo, Finland) and PerioCheck (CollaGenex Pharmaceuticals, Newton, PA, USA) tests, which are quick to apply chairside and provide reliable oral bacteria test results. The *S. mutans* levels in the parent–child pairs were measured from plaque and the tongue and scored from 0 to 3, indicating bacteria growth of ≤10^4^, <10^5^, 10^5^–10^6^, and >10^6^ Colony Forming Unit/mL (CFU/mL), respectively. The rapid chairside diagnostic test kit for neutral proteases in periodontics, the PerioCheck test, measures neutral protease activity within the gingival crevicular fluid, such as elastases, proteinases, and collagenases of periodontopathic bacterial origin. The PerioCheck test results were scored between 0 and 2 for each participant by comparing the intensity of staining with the color chart provided by the manufacturer, which represents the level of neutral protease in the sample.

### 2.4. Study Analysis

The present study is a retrospective analysis of collected clinical and microbiological data from parent-child pairs. All analyses were performed using the software SPSS 17.0 for Windows (IBM Corporation, Armonk, New York, U.S.). The Kendall rank correlation coefficient was used to measure the association between DMFT and dmft indices, PCR score, plaque and tongue *S. mutans* levels, periodontal health classification score, PerioCheck score, periodontal pocket depth, and gingival index in parents and children. The model was built according to a C4.5 DT.

#### C4.5: A DT-Based Classifier

The DT model in this study was generated by the most widely used algorithm for DT induction (C4.5), which is a statistical classifier developed by Quinlan [14]. The DT structure consists of a leaf or a class value and a decision node, which specifies a test to be conducted on one of the attributes. In our study, the collected data of clinical and microbiological examination comprised 12 attributes (Table 1), including (1) parent’s PCR score, pocket depth, tongue and plaque *S. mutans* level, periodontal health classification, PerioCheck score, gingival and DMFT indices; (2) child’s PCR score, PerioCheck score, tongue and plaque *S. mutans* level. Table 1 presents the description of each attribute. At each node of the tree, C4.5 chooses the attribute of the data that most effectively splits its set of samples into subsets according to the splitting criterion (normalized information gain). The attribute with the highest normalized information gain is chosen to make the decision.

For variables with several outcomes, we used a weighted sum of the log_2_(*p_j_*), with weights equal to the outcome probabilities, resulting in the following formula:

(1) $$Entropy\left(X\right)=\underset{j=1}{\sum }p_{j}\log_{2}\left(p_{j}\right)$$

where *p_j_* is the probability of the occurrence of the number of class and represents the proportion of *X* that belongs to the class *j*. C4.5 uses the following concept of entropy: Suppose that candidate attribute (*S*) can divide the training set (*T*) into the subsets *S*_1_, *S*_2_,…, *S_n_*. The mean information requirement can be measured as the weighted sum of entropies for each partitioned subset, as follows:

(2) $$H_{s}\left(T\right)=\overset{k}{\underset{i=1}{\sum }}P_{i}H_{s}\left(T_{i}\right)$$

where *P_i_* represents the proportion of records in each partitioned subset *i*. The information gain of the attribute *Gain* (*S*) is relative to the collection of the examples *X* and can be defined as:

(3) $$Gain\;\left(S\right)=Entropy\;\left(X\right)-H_{s}\left(T\right)$$

At each node of the tree, C4.5 chooses the highest information gain Equation (3) corresponding to the suitable split and determines the threshold value for the attribute of the data that most effectively differentiates the target variable, thus most effectively splitting its set of samples and reducing classification errors. The splits are determined by considering all possible dichotomizations of all of the available predictor variables. In the present study, an attribute was not retested further down the tree because there is one branch for each possible value. For continuous attributes (i.e., DMFT and probing depth), the test at a node determined whether its value was higher or lower than a predetermined constant.

## 3. Results

Table 2 presents the distribution of periodontal status and caries indices from oral examination findings in 30 parent–child pairs. The median of the DMFT index in parents was 13.0 (interquartile range (IQR) = 6.0) and the PCR score was 87.45 (IQR = 20.25). In the periodontal assessment, the parents exhibited a median pocket depth of 1.87 (IQR = 0.62) and gingival index of 1.37 (IQR = 0.59). Children exhibited a median dmft index of 9.00 (IQR = 8.0) and PCR score of 94.55 (IQR = 20). Periodontal examination was not performed in children; thus, periodontal findings are not described. Microbiological examination of *S. mutans* in plaque, demonstrated that 26.67% of parents scored 0, 23.33% scored 1, 16.67% scored 2, and 33.3% scored 3. Tests for *S. mutans* on the tongue revealed that 20% of parents scored 0, 30% scored 1, 26.67% scored 2, and 23.33% scored 3. Among children, 36.66% reported a high level of *S. mutans* with a plaque score of 2 or 3. A 43.33% of children presented with high growth level of *S. mutans* in tongue with a score of 2 and 3. Periodontal examination results showed that 60% of parents had healthy periodontal status, 23.33% had Class 1, 6.67% had Class 2, and 10% had Class 3 periodontal status. All examined children were periodontally healthy. PerioCheck results indicated that 46.67% of parents scored 1 and 26.67% scored 0 or 2, and among the children, 70% scored 1 and 30% scored 0.

Table 3 shows the correlation between periodontal status, caries indices, and oral test examination findings in parents and children. Parents’ DMFT index positively correlated with their *S. mutans* level on the tongue (*p* = 0.27). Among parents, a positive correlation was observed between *S. mutans* levels in plaque and the tongue (*p* = 0.57). More severe periodontal classification (*p* = 0.30) and deeper periodontal probing depth (*p* = 0.33) was observed in parents with higher periodontal pathogen levels than their counterparts. Moreover, the periodontal classification became more severe as periodontal probing depth increased in parents (*p* = 0.65). The gingival index in parents was associated with their PCR score (*p* = 0.22) and the *S. mutans* level in plaque (*p* = 0.34). In children, *S. mutans* concentration on the tongue was associated with the dmft index (*p* = 0.23) and *S. mutans* in plaque (*p* = 0.68). Moreover, the periodontal pathogen number was associated with *S. mutans* concentrations in plaque (*p* = 0.32) and on the tongue (*p* = 0.38) among children.

**Figure 1:**
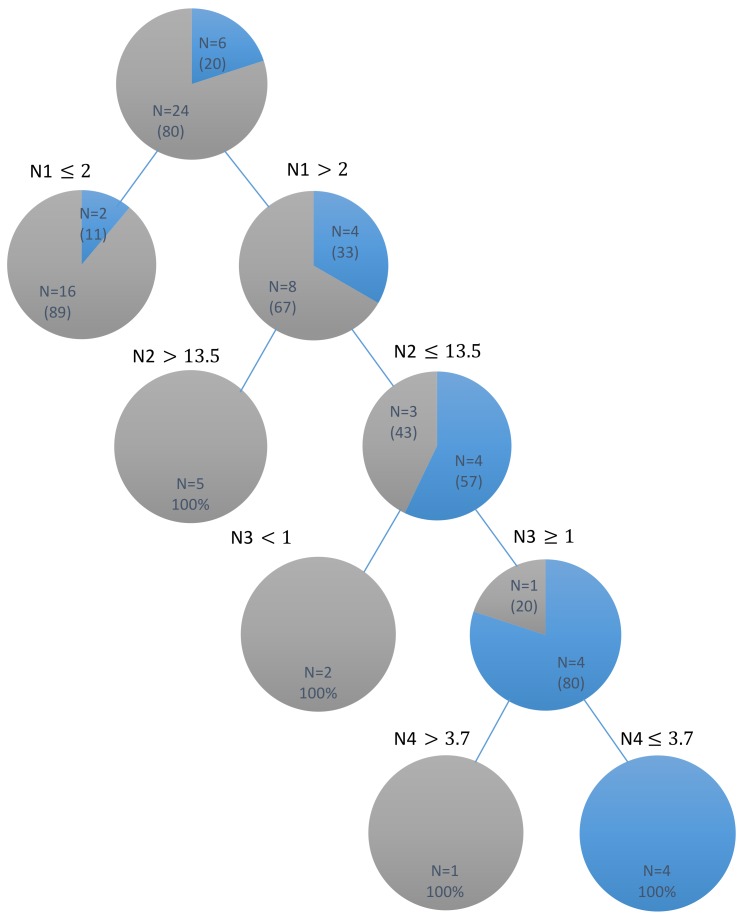
DT (decision tree) for the dental caries outcome in children identified by their parents’ periodontal clinical and microbiological outcomes. 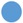 children with dmft ≤3;  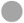 children with dmft > 3; Four nodes of DT were determined according to: N1: parent’s periodontal classification; N2: parent’s DMFT score; N3: parent’s Perio-Check test results; N4: parent’s pocket depth.

The DT model of parents’ oral health status and dental caries in children is shown in Figure 1. The independent variables included in this model to identify the outcome variables of children’s dmft indices are listed in Table 1. The DT revealed that 88.8% of children, whose parents had a periodontal classification ≤2, had dmft indices >3. Among children whose parents had a periodontal classification >2, 41.6% had dmft indices >3 when their parents’ DMFT index was >13.5. Conversely, two children, whose parents had a periodontal classification >2, PerioCheck score of 1, and DMFT index <13.5, were identified to have a dmft index >3. Parent’s periodontal pocket depth was selected as the final decision node to identify children with dmft indices <3, and the results showed that four children with a dmft index <3 had parents with a periodontal pocket depth ≤3.7, PerioCheck score ≥1, DMFT index <13.5, and periodontal classification >2. Table 4 presents the sensitivity and specificity for each variable identified by the DT for the prediction of caries outcome in children. This DT model had a prediction accuracy of 93.33% for the study sample.

## 4. Discussion

Caries-risk data in dentistry are still not sufficient to quantitate the models. Caries-risk assessment models currently include a combination of factors, such as diet, fluoride exposure, a susceptible host, and microflora that interplay with a variety of social, cultural, and behavioral factors. Although the best tool to predict future caries is past caries experience, it is not particularly useful in young children due to the importance of determining caries risk before the disease is manifest [19].

Decision trees have many advantages. One important advantage is the ease of interpretation despite the tree being complex, involving a large number of splits and nodes. This cross-sectional study does not enable inferences regarding causal factors. However, this design does enable the evaluation of the associated factors of dental caries in children. The decision tree builds classification or regression models in the form of a tree structure, which is a reliable and effective decision making technique that provides high classification accuracy [20].

In this cross-sectional study, we proposed a pilot model that used DT analysis on high-caries risk group in preschool children by using clinical and microbiological data obtained from parent–child pairs. Parental periodontal status variables were major contributing factors in addition to their previous caries experience in categorizing the children into high and low caries groups. A DT model established two caries groups of children in this study: the high-caries group consisted of patients with a dmft index >3 and the low-caries group had a dmft index <3. The factors included in the DT to identify children within these groups included both the parent and children’s PCR scores, *S. mutans* levels in the tongue and plaque, PerioCheck scores, parent’s periodontal pocket depth, CPI index, and gingival and DMFT indices. In the DT, the descending order of these factors (parents’ CPI index, DMFT, PerioCheck score, and probing depth) was crucial in identifying low-caries patients. The DT model showed that an increased parental periodontal pathogen level and a probing depth ≤3.7 mm were associated with low caries in children; however, these factors are inadequate to evaluate individual parent’s periodontal disease status. Thus, the periodontal health classification (CPI index) used in our study provides insight into the periodontal disease levels of participants and can be used to evaluate their treatment needs. The threshold value of the CPI index on the node of DT model was 2, which indicates that parents of low-caries group children were classified as grade 2 and above. According to the classification, parents who had calculus with plaque determined through probing required oral hygiene improvement for plaque control in addition to professional scaling and root planning. 

**Figure A1:**
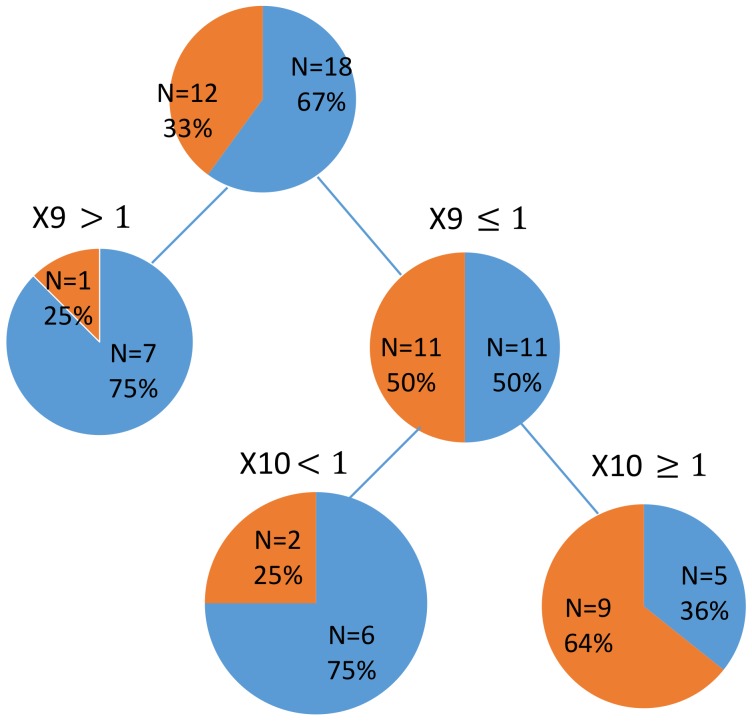
DT for the dental caries outcome in children identified by their parents’ clinical and microbiological data. (periodontal condition was eliminated, Exclude x8 and x3); 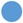 children with dmft ≤10;  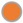 children with dmft > 10; X9: Perio Check (parent); X10: Perio Check (Children).

Periodontal score and periodontal pathogen score were the major influential factors in DT analysis. Since DT analysis was a classification tool in the study, periodontal condition was not a factor leading to caries risk. The proper explanation of this result is discussed as follows. Periodontal disease and dental caries are the most common diseases of humans and the main cause of tooth loss. Both diseases can lead to nutritional compromise and negative impacts upon self-esteem and quality of life. As complex chronic diseases, they share common risk factors, such as a requirement for a pathogenic plaque biofilm, yet they exhibit distinct pathophysiology. Multiple exposures contribute to their causal pathways, and susceptibility involves risk factors that are inherited (e.g., genetic variants) and those that are acquired (e.g., socio-economic factors, biofilm load or composition, smoking, carbohydrate intake). Identification of these factors is crucial in the prevention of both diseases as well as in their management [21]. When we excluded clinical periodontal condition, PerioCheck score proved to be the factor forming the DT for children’s caries experience (Appendix A, Figure A1).

The oral cavity is a complex ecology consisting of more than 700 bacterial species [22]. However, when the balance among species is compromised because of the environment, rearrangement of normal flora can lead to either cariogenic or periodontopathic plaque [23,24]. The pathogens involved and the pathophysiology of these infections are dissimilar because periodontal disease involves a group of bacteria that are metabolic opposites of those involved in cariogenesis. Dental caries reflects an acellular response of the teeth to the bacterial challenge, whereas periodontal disease reflects a cellular inflammatory response of the gingiva and surrounding connective tissue to subgingival bacterial accumulation. Moreover, subgingival bacteria initially colonize the supragingival plaque and then move beneath the gingival margin where the pressure of oxygen tension is lower, causing them to become anaerobic. Although dental caries and periodontal disease are different infections, the results of this study indicate that a relationship existing between these two infections within a family is likely. In addition, the results from this sample revealed a decrease in children’s dmft index scores with an increasing caregiver’s probing depth [5]. Despite the mechanism behind these factors being unclear, it may be related to the complex influences of environment and genetics regarding oral health, specifically the ability to host bacteria. However, no evidence is available that supports familial correlation in the ability to host bacteria within supragingival and subgingival plaque because of a lack of research in this field.

The multifactorial nature of the disease [25,26] is suggested by the widely established knowledge that susceptibility to caries may be affected by a combination of environmental, behavioral, and biological factors, including demographics, dietary behaviors, bacterial challenge, oral hygiene, fluoride intake and exposures, salivary composition and flow rate, tooth positional and morphological features, and genetic components contributing to enamel formation, saliva composition, and immune responses [27,28,29]. Although the complexity of the disease is known, it is not possible to assess all the caries risk factors when assessing a child’s caries risk in a clinical practice setting. Moreover, in most caries prediction models that are currently used, the first included predictor variable is past caries experience, followed by pathogenic bacterial levels [30]. Thus, our main goal was to investigate whether a DT can identify caries risk groups from the given clinical data, which can easily be found on dental charts. Our findings indicate the effect of parental periodontal health status and caries index on children’s caries experience. According to the DT results, preschool children, whose caregiver had higher periodontal CPI index score and needed intensive oral health care education, were in need of further caries risk assessment implementation. Those children could be classified as an intensive care group. 

Decision trees give good predictive accuracy and tolerate missing data in deployment. However, the reliability of the information in the decision tree depends on feeding the precise internal and external information at the onset. Even a small change in input data can at times cause large changes in the tree. Another fundamental flaw of decision tree analysis is that the decisions contained in the tree are based on expectations, and irrational expectations can lead to flaws and errors. The decision tree concept and automatic learning can be successfully used in real world situations and constrained with real world limitations, but they should be used only with the guidelines of appropriate medical experts [31].

Currently used caries prediction models are commonly based on either logistic discriminant analysis, logistic regression analysis, multiple regression analysis and classification, or regression trees [26]. However, the predictive validity of the models depends heavily on the caries prevalence and characteristics of the population for which they were designed. No combination of risk indicators has been consistently reported as an accurate predictor when applied to various populations across different age groups [32]. Therefore, efforts to develop a single, inclusive, and accurate model for caries risk assessment have been unsuccessful, particularly for young children. DT analysis may be suitable and accurate for the classification of grouping high-risk potential models when applied to various populations because it is capable of choosing different numbers of variables among diverse population groups. DT models are more assumption-free, handle missing data more efficiently, may be more population-specific, and appear to be more analytically parsimonious when compared with linear discriminant analysis and logistic regression analysis [33]. Unlike logit models, DT models are robust to outliers, do not require specific data transformations or hierarchical interaction specification, and can be easily interpreted clinically [34]. These are some of the numerous compelling reasons for proposing the use of tree-structured classification methods in caries risk assessment. In the present study, the accuracy level of the DT model reached 93.33% and the sensitivity and specificity level reached 160% for each indicator (Table 4).

Detecting high-caries risk potential group is useful not only in planning the clinical decision-making process and determining the timing of recall appointments but also in identifying the target population for further intervention. Since caries risk indicators likely differ with age group and population [35,36,37], it may be insufficient to include the same risk indicators for individual risk assessments that clinicians apply on a daily basis. Therefore, there is an increasing interest in developing suitable caries risk models for community-based settings to enhance identification of high-risk groups to enhance preventive care. A previous study conducted in a Scottish community developed a caries risk model for potential use in a particular community setting, structured in a DT format and enhanced with traditional statistical approaches to identify high-caries risk individuals at 1 year of age [38]. Accurate risk prediction for an individual child would make it possible to direct the most intensive preventive services to those in need. Therefore, preventive interventions must be targeted to various risk groups. A high-risk strategy is adequate for dental practitioners focusing on individuals with the highest risk of caries. This strategy is matched individually and increases the likelihood of a cost-effective use of resources [39]. A recent study demonstrated that preventive individualized intervention programs providing oral health education to low caries risk patient groups are not beneficial and recommended against fluoride varnish application every 6 months for children younger than 3 years [40].

Several limitations in the present study must be considered. The number of participants may limit the representativeness of the sample. Thus, additional investigations with larger samples are required to apply the DT method to assess caries risk within population. The cross-sectional design does not enable inferences regarding causal factors. Moreover, adequate methods should be employed following the guidelines of the STROBE (Strengthening the Reporting of Observational studies in Epidemiology) statement. Longitudinal studies are needed to evaluate the long-term effects of dental caries in preschool children. Additionally, individual variables, such as tooth brushing patterns, salivary flow, dietary habits, caregivers’ education, socioeconomic status, and use of fluoride were not addressed in the present investigation. Therefore, further studies that include such variables using decision tree analysis are needed which could assist in the establishment of oral health policies.

## 5. Consent to Publish

Written informed consent for publication of parents’ and children’s clinical details was obtained from the parent/guardian of the patient. A copy of the consent form is available for review by the Editor of this journal.

## 6. Conclusions

The present cross-sectional study identified a pilot model of high-caries risk children from their parents’ dental caries and periodontal health data. Parents’ periodontal health status might serve as another variable in high-risk potential groups for preschool children. The decision tree analysis results could contribute to decision-making regarding the establishment of parents’ oral health priorities for preschool children. Routinely questioning patients about their family history of periodontal diseases and caries is recommended. 

## Figures and Tables

**Table 1:** Attributes included in the DT (decision tree).

X: Variables Included in DT	Item	Description
X¹	PCR (parent)	Parent’s PCR score
X²	PCR (child)	Child’s PCR score
X³	PD	Parent’s pocket depth (mm)
X⁴	Parent SM (tongue)	Parent’s *S. mutans* level (tongue)
X⁵	Parent SM (plaque)	Parent’s *S. mutans* level (plaque)
X⁶	Child SM (tongue)	Child’s *S. mutans* level (tongue)
X⁷	Child SM (plaque)	Child’s *S. mutans* level (plaque)
X⁸	CPI Index	Parent’s periodontal classification
X⁹	PerioCheck (parent)	Parent’s PerioCheck results
X¹⁰	PerioCheck (child)	Child’s PerioCheck results
X¹¹	GI	Parent’s gingival index
X¹²	DMFT	Decayed, missing, filled (because of caries only) permanent teeth
Y: outcome variable		
Y¹	dmft index	Decayed, missing, filled (because of caries only) primary teeth

**Table 2:** Distribution of periodontal status and caries index scores in parent–child pairs.

	Parent		Child	
	Median	IQR	Median	IQR
DMFT/dmft	13.00	6.00	9.00	8.00
PCR	87.45	20.25	94.55	20.00
Pocket depth	1.87	0.62	-	-
Gingival index	1.37	0.59	-	-
	*N*	%	*N*	%
SM (plaque)				
0	8	26.67	9	30.00
1	7	23.33	10	33.33
2	5	16.67	4	13.33
3	10	33.33	7	23.33
SM (tongue)				
0	6	20.00	13	43.33
1	9	30.00	4	13.33
2	8	26.67	6	20.00
3	7	23.33	7	23.33
Periodontal classification				
0	18	60.00	30	100.00
1	7	23.33		
2	2	6.67		
3	3	10.00		
PerioCheck				
0	8	26.67	9	30.00
1	14	46.67	21	70.00
2	8	26.67		

IQR: interquartile range; DMFT: decayed, missing, filled permanent teeth; PCR: plaque control record; SM: *Streptococcus mutans.*

**Table 3:** Kendall rank correlation coefficient between periodontal status and caries index strata for parents and children.

**a. Parents**							
	**PCR**	**SM (Plaque)**	**SM (Tongue)**	**Periodontal Classification**	**Perio Check**	**Pocket Depth**	**Gingival Index**
DMFT	0.06	0.17	0.27 ^+^	−0.24	0.18	−0.10	0.11
PCR		0.23	0.19	0.11	0.00	0.13	0.22 ^+^
SM (plaque)			0.57 ***	0.05	0.20	0.12	0.34 *
SM (tongue)				−0.16	0.07	−0.09	0.13
Periodontal classification				1.00	0.30 ^+^	0.65 ***	0.15
PerioCheck						0.33 *	0.18
Pocket depth							0.19
**b. Children**							
	**PCR**		**SM (Plaque)**	**SM (Tongue)**		**PerioCheck**	
dmft	0.05		0.14	0.23 ^+^		0.16	
PCR			0.24	0.10		−0.04	
SM (plaque)				0.68 ***		0.32 ^+^	
SM (tongue)						0.38 *	

*** *p* < 0.001 * *p* < 0.05; ^+^ 0.1 > *p* > 0.05; SM: *Streptococcus mutans.*

**Table 4:** Sensitivity and specificity for variables identified using the DT for the prediction of caries outcome (dmft) in children.

	Sensitivity (%)	Specificity (%)
N1 (parent’s periodontal classification)	100.0	66.67
N2 (parent’s DMFT score)	100.0	100.0
N3 (parent’s PerioCheck results)	100.0	100.0
N4 (parent’s periodontal pocket depth)	100.0	100.0
